# Phylogenetic analysis of plant multi-domain SEC14-like phosphatidylinositol transfer proteins and structure–function properties of PATELLIN2

**DOI:** 10.1007/s11103-020-01067-y

**Published:** 2020-09-11

**Authors:** Karolin Montag, Jannik Hornbergs, Rumen Ivanov, Petra Bauer

**Affiliations:** Institute of Botany, Heinrich Heine University, 40225 Düsseldorf, Germany

**Keywords:** SEC14, PATELLIN, Membrane, GOLD domain, Liposome, Phosphatidylinositides

## Abstract

**Key message:**

SEC14L-PITPs guide membrane recognition and signaling. An increasingly complex modular structure of SEC14L-PITPs evolved in land plants compared to green algae. SEC14/CRAL-TRIO and GOLD domains govern membrane binding specificity.

**Abstract:**

SEC14-like phosphatidylinositol transfer proteins (SEC14L-PITPs) provide cues for membrane identity by exchanging lipophilic substrates, ultimately governing membrane signaling. Flowering plant SEC14L-PITPs often have modular structure and are associated with cell division, development, and stress responses. Yet, structure–function relationships for biochemical–cellular interactions of SEC14L-PITPs are rather enigmatic. Here, we evaluate the phylogenetic relationships of the SEC14L-PITP superfamily in the green lineage. Compared to green algae, land plants have an extended set of SEC14L-PITPs with increasingly complex modular structure. SEC14-GOLD PITPs, present in land plants but not Chara, diverged to three functional subgroups, represented by the six PATELLIN (PATL) proteins in Arabidopsis. Based on the example of Arabidopsis PATL2, we dissect the functional domains for in vitro binding to phosphoinositides and liposomes and for plant cell membrane association. While the SEC14 domain and its CRAL-TRIO-N-terminal extension serve general membrane attachment of the protein, the C-terminal GOLD domain directs it to the plasma membrane by recognizing specific phosphoinositides. We discuss that the different domains of SEC14L-PITPs integrate developmental and environmental signals to control SEC14L-PITP-mediated membrane identity, important to initiate dynamic membrane events.

**Electronic supplementary material:**

The online version of this article (10.1007/s11103-020-01067-y) contains supplementary material, which is available to authorized users.

## Introduction

Biological membranes define two milieus by selectively regulating the exchange of substances and flow of information. Membrane proteins dynamically interact with membrane lipids to monitor and elicit regulatory steps at membranes and in between different membranes, such as during secretion and endocytosis.

One protein family with membrane recognition and signaling properties is the SEC14-like phosphatidylinositol transfer protein (SEC14L-PITP) superfamily. SEC14L-PITPs, first identified in yeast (*Saccharomyces cerevisiae*) by screening for secretory mutants, share their characteristic SEC14 domain, also known as CRAL-TRIO domain (Novick et al. 1980). The SEC14 domain of yeast prototype Sec14p starts with three alpha helices, sufficient for stable Golgi membrane association (Sha et al. 1998; Skinner et al. 1993). Not all SEC14 domains contain this alpha-helical region, named CRAL-TRIO-N-terminal extension (CTN) (Saito et al. 2007a). The SEC14 domain acts as a pocket with lid for binding, transferring and exchanging different types of single lipophilic substrates between membranes, e.g. phosphatidylinositol (PI), phosphatidylinositol phosphates/phosphoinositides (PIPs) and phosphatidylcholine (PC) (Bankaitis et al. 1990; Cleves et al. 1991). This non-vesicular transport and heterotypic exchange of chemically different substances affect membrane identity, e.g. through exchanging PC against PIPs, or by recruitment of a PI-kinase (de Campos and Schaaf 2017; Schaaf et al. 2008). Resulting signals affect downstream cellular events, such as lipid kinase efficiency and vesicle formation (Bankaitis et al. 2010). Heterotypic exchange can also occur against tocopherol and retinal, altering membrane characteristics (Kono et al. 2013; Panagabko et al. 2003; Saito et al. 2007a). SEC14L-PITPs act in complex physiological processes. Human diseases and defects in plant development, and stress response have been associated with mutations in SEC14L-PITP-encoding genes (Curwin and McMaster 2008; Zhou et al. 2019).

How SEC14L-PITP activities are regulated in the cell is a question still under investigation. However, it is discussed that the modular composition and specialization of protein functions of the large SEC14L-PITP families in higher eukaryotes serve to integrate the SEC14L-PITP activities in the different cellular contexts.

While yeast has only six members with only the fundamental SEC14 or CTN-SEC14 domains, higher eukaryotes, e.g. humans or the flowering plant Arabidopsis (*Arabidopsis thaliana*), not only have high numbers of SEC14L-PITPs, but also many of them with modular complex composition. These complex SEC14L-PITPs carry at their C- or N-terminus additional stretches, mostly with recognizable domains, also found in other types of proteins (Anantharaman and Aravind 2002; Saito et al. 2007a).

Arabidopsis has six multi-domain SEC14L-PITPs, named PATELLINs (PATLs), containing an additional Golgi dynamics (GOLD) domain at their C-terminus (Peterman et al. 2004). GOLD domain proteins are involved in Golgi function and vesicle trafficking, and this domain might play a role in mediating protein–protein interactions with putative cargo proteins as well as mediating protein–lipid interactions (Anantharaman and Aravind 2002; Carney and Bowen 2004). Arabidopsis PATL3 recruitment to the plasma membrane (PM) is dependent on an interaction with exocyst component EXO70A1 via the GOLD domain (Wu et al. 2017). The exocyst complex is involved in cytokinesis and establishment of cell polarity (Fendrych et al. 2013; He and Guo 2009). PATL proteins also exhibit a large, often acidic N-terminal region with variable amino acid composition, a high amount of charged amino acids [e.g. (K)KE(E); (EE)EK repeats], coiled coil and PxxP motifs (Diella et al. 2008; Neduva and Russell 2006; Peterman et al. 2004).

Plant mutant analysis and protein localization suggest that PATLs are important for cell division, polarity and patterning (Peiro et al. 2014; Suzuki et al. 2016). Multiple homozygous gene knock-outs have drastic developmental defects in Arabidopsis. The plants lack proper polarization of the auxin transporter PIN-FORMED1 (PIN1) (Tejos et al. 2017). PATL1 localizes to the developing cell plate and is able to bind PI and PIPs, preferring PI(5)P, PI(3)P and PI(4,5)P_2_ on lipid strips (Peterman et al. 2004). PATL2 binds all PIPs but not PI and localizes to the PM and the developing cell plate during cytokinesis in roots (Suzuki et al. 2016; Tejos et al. 2017). Furthermore, environmental response phenotypes are reported, indicating that PATL proteins play a role in the proper functioning of organs. PATL1 interacts with the Ca^2+^ sensor Calmodulin-4 (CaM4), and PM Na^+^/H^+^ antiporter SALT OVERLY-SENSITIVE1 (SOS1), and contributes to the plant tolerance to cold and salt stress (Chu et al. 2018; Zhou et al. 2018). PATL1 negatively influences reactive oxygen species formation, linking it to cellular damage protection under stress (Zhou et al. 2018). PATL3 and PATL6 disturb alfalfa mosaic virus movement by interaction with a plasmodesmata targeting movement protein (Peiro et al. 2014).

Here, we performed a phylogenetic analysis of the SEC14L-PITP family in the green lineage to assess whether the complexity of SEC14L-PITPs increased during land plant evolution, as was the case in the lineage leading to animals. Based on the example of Arabidopsis PATL2 protein, we dissect the modular structure of a multi-domain SEC14-GOLD protein to investigate the contribution of individual domains to lipid binding and membrane association.

## Materials and methods

### Phylogenetic analysis and alignments

SEC14 protein sequences were identified via Phytozome v12.1 (https://phytozome.jgi.doe.gov) and protein BLASTp (https://blast.ncbi.nlm.nih.gov/Blast.cgi?PAGE=Proteins) using as query yeast Sec14p and the SEC14 domain of AtPATL2, and via InterPro using the CRAL-TRIO domain. Sequences were retrieved from selected species of green algae, a moss, a lycophyte, and of selected flowering plants, and analyzed for the presence of multiple domains. The positions of domains were determined using NCBI Conserved Domain Search NIH (http://www.ncbi.nlm.nih.gov/Structure/cdd/wrpsb.cgi), PROSITE (https://prosite.expasy.org/) and InterPro (https://www.ebi.ac.uk/interpro). InterPro domain accession numbers are GOLD-IPR009038, SEC14/CRAL-TRIO-IPR001251, CTN-IPR011074, GDAP2/Macro domain-IPR035793, DDT domain-IPR018501, PHD domain-IPR019787, WHIM1 domain-IPR028942, WHIM2 domain-IRO28941. Nodulin and nodulin-like domains were defined according to Kapranov et al. (2001) and Vincent et al. (2005).

Phylogenetic trees were generated as described by Ivanov and Bauer (2017) using the https://www.phylogeny.fr server.

### Accession numbers

*Arabidopsis thaliana* TAIR10: At4g344580 (AtSfH1); AT2G21540 (AtSfH3); At4G36490 (AtSfH12); At1g72150 (PATL1); At1g22530 (PATL2); At1g72160 (PATL3); At1g30690 (PATL4); At4g09160 (PATL5); At3g51670(PATL6); At4g39180; At1g01630; At1g05370; At1g14820; At1g19650; At1g22180; At1g55840; At1g75170; At1g75370; At2g15670; At2g16380; At2g18180; At2g21520; At3g22410; At3g24840; At3g46450; At4g08690; At4g36640; At4g39170; At5G47730; At5g56160; At5g63060; At1g69340; At4g35750; At3g10210; At1g55690.

*Chlamydomonas reinhardtii* v5.5: Cre10.g444250; Cre12.g503950; Cre12.g527050.t1.2; Cre10.g448051; Cre02.g141950; Cre03.g166201; Cre02.g147800; Cre02.g101200; Cre17.g718100; Cre17.g703200; Cre11.g467563.

*Volvox carteri* v2.1: Vocar.0070s0030; Vocar.0004s0276; Vocar.0005s0399; Vocar.0008s0014; Vocar.0036s0128; Vocar.0037s0062.

*Chara braunii* (Nishiyama et al. 2018): CBR_g84; CBR_g29298; CBR_g36387; CBR_g31494; CBR_g38007; CBR_g39624; CBR_g4074.

*Marchantia polymorpha* v3.1: Mapoly0098s0038; Mapoly0054s0137; Mapoly0008s0106; Mapoly0027s0072; Mapoly0054s0139; Mapoly0064s0114; Mapoly0091s0075; Mapoly0245s0002; Mapoly0114s0025; Mapoly0030s0043; Mapoly0153s0026.

*Selaginella moellendorffii* v1.0: 439610; 271658; 114753; 11806; 168470; 121430; 93038; 92905; 91570; 43741; 65145; 90159; 89782; 91055; 77842; 22919.

*Solanum lycopersicum* iTAG2.4: Solyc07g066090; Solyc10g053900; Solyc04g082050; Solyc04g040200; Solyc11g051160; Solyc08g078680; Solyc11g040280; Solyc01g109870; Solyc01g005270; Solyc02g070210; Solyc01g005290; Solyc01g109860; Solyc03g118150; Solyc02g083250; Solyc10g053970; Solyc10g005740; Solyc01g005280; Solyc05g054570; Solyc01g005260; Solyc12g089130; Solyc06g075980; Solyc05g012610; Solyc09g015080 (SITBP); Solyc11g027880; Solyc09g025230; Solyc04g080690; Solyc06g064940; Solyc09g060090; Solyc11g012790; Solyc03g112640; Solyc07g065700; Solyc04g005490.

*Zea mays* PH207 v1.1: Zm00008a000784; Zm00008a007108; Zm00008a021866; Zm00008a034613; Zm00008a000376; Zm00008a008524; Zm00008a018020; Zm00008a031256; Zm00008a016003; Zm00008a033937; Zm00008a031138; Zm00008a013174; Zm00008a027397; Zm00008a019107; Zm00008a032518; Zm00008a032332; Zm00008a025849; Zm00008a025849; Zm00008a039685; Zm00008a008448; Zm00008a022847; Zm00008a022585; Zm00008a025654; Zm00008a018864; Zm00008a017887; Zm00008a003138; Zm00008a012744; Zm00008a037114; Zm00008a037115; Zm00008a019021; Zm00008a004286; Zm00008a022784; Zm00008a012865; Zm00008a016847; Zm00008a028031; Zm00008ă80; Zm00008a005640; Zm00008a025640; Zm00008a026335; Zm00008a009102; Zm00008a017141; Zm00008a033936; Zm00008a012432; Zm00008a013571; Zm00008a025498; Zm00008a028943; Zm00008a008572; Zm00008a033935; Zm00008a032805; Zm00008a034926; Zm00008a020872; Zm00008a020873; Zm00008a014049; Zm00008a021294.

### Visualization of gene expression and gene co-expression analysis

Expression data for Arabidopsis *PATL1*, *PATL2*, *PATL3*, *PATL4* and *PATL6* genes were obtained using the ePlant Heatmap Viewer module (https://bar.utoronto.ca/eplant/) (Waese et al. 2017). Co-expression networks were built using the Network Drawer module via ATTED-II version 9.2 (https://atted.jp/) (Obayashi et al. 2018). The gene ontology (GO) feature of ATTED-II was used for GO term enrichment.

### Generation of recombinant vectors

Full-length Arabidopsis *PATL2* and derived deletion mutants (Table S2) were generated as follows: The *PATL2* coding sequence was amplified from cDNA, obtained from wild-type Col-0 roots, using primers PATL2B1F and PATL2B2stopR (Table S3) and transferred via Gateway cloning into pDONR207 (BP reaction, Life Technologies). This plasmid was used as a template for generating all different *PATL2* deletion mutant coding sequences by PCR (Table S3 for primers), subsequently transferred into pDONR207. Then, *PATL2* and deletion-mutant forms were transferred into pH7WGY2 vector via Gateway cloning (LR reaction, Life Technologies), allowing constitutive expression of N-terminally tagged YFP proteins in plant cells via pCaMV35S (Karimi et al. 2002). The pDONR plasmids served as templates for generating recombinant pET-52b (+) plasmids (Novagen) with PATL2 and deletion-mutant inserts, using restriction-ligation cloning with *Bam*HI and *Not*I restriction enzymes, allowing Strep-tagged protein expression in bacteria.

### Transient tobacco epidermis transformation and confocal microscopy

*Rhizobium radiobacter* strain C58C1(pTiB6S3ΔT)^H^ containing recombinant pH7WGY2 with PATL2 or PATL2-mutant inserts was used for transforming tobacco (*Nicotiana benthamiana)* by leaf infiltration, as previously described (Hotzer et al. 2012). Overnight *R*. *radiobacter* cultures in YEB medium were pelleted and resuspended to an OD_600_ of 0.4 in tobacco infiltration solution (2 mM NaH_2_PO_4_, 50 mM MES, 0.5% Glucose) supplemented with 100 μM acetosyringone. The suspension was infiltrated into young tobacco leaves. After 24–48 h, leaves were used for confocal microscopy. For co-localization studies, the PATL2 vector-containing bacteria were mixed with bacteria containing plant vectors for expression of PM marker AHA1-mRFP (Caesar et al. 2011) and ER marker mOFP-HDEL (Batistic et al. 2008).

Confocal images of fluorescent signals were collected using a LSM780 system (Zeiss). YFP signals were excited at 514 nm, and emission was detected at 520–550 nm. mRFP and mOFP were excited at 561 nm, and emission was detected at 580–630 nm.

### Protein expression and purification with the Strep-tag®/Strep-Tactin® system

BL21 (DE3) pLysS-containing pET-52b(+) vectors with recombinant PATL2 or PATL2 deletion mutants were grown in 50 ml LB (100 µg/ml ampicillin, 34 µg/ml chloramphenicol, OD_600_ of 0.08). The temperature was lowered from 37 to 30 °C when the culture reached an OD_600_ of 0.3–0.4. Protein expression was induced 30 min after the temperature shift with 1 mM isopropyl-ϐ-d-thiogalactopyranosid (IPTG) and cells were harvested 3 h later. StrepII-tagged protein of interest was purified using the Strep-tag®/Strep-Tactin® system (IBA Lifesciences) according to the manufacturer’s protocol for Strep-Tactin® Macroprep® or via Strep-Tactin®XT Superflow® cartridge using ÄKTA prime plus (GE Healthcare Life Sciences). Protein expression and purification were assessed following standard SDS–polyacrylamide gel electrophoresis, either followed by Coomassie Brilliant Blue staining or by blotting on Amersham™ Protran™ 0.2 µm nitrocellulose membranes (GE Healthcare Life Sciences) and affinity detection via enhanced chemiluminescence (GE Healthcare Life Sciences) of StrepII-tagged protein using Strep-Tactin®-horseradish peroxidase (HRP) conjugate (Iba Lifesciences).

### Protein–lipid overlay assays

Membrane Lipid Strips™ or PIP Strips™ (Echelon Biosciences) were blocked with 3% (w/v) BSA in TBST (150 mM NaCl, 2.7 mM KCl, 24.7 mM Tris–HCl, 0.05% v/v Tween-20, pH 7.4) for 2 ½ h at room temperature in a Petri plate. The protein–lipid overlay incubation was performed with 2 ml Strep-tagged protein (0.5 µg/ml protein in 3% (w/v) BSA in TBST) at 4 °C overnight. The strip was washed five times for 8 min with TBST and incubated with 1:1000 diluted custom-made α-PATL2-1 IgG antibody, generated against the peptide TKKEETPVAPAPVEC (GenScript), in 3% (w/v) BSA in TBST for one hour at room temperature. The strip was washed four times for ten minutes with TBST and incubated for 1 h with 1:2000 Goat anti-rabbit IgG-HRP-conjugated antibody (Agrisera) in 3% (w/v) BSA in TBST. After four washing steps with TBST for 10 min, the protein–lipid binding was detected by enhanced chemiluminescence (GE Healthcare Life Sciences) and quantified.

For signal quantification of lipid dot binding, the Multiplex Band Analysis feature of AlphaView®Software (ProteinSimple) was used. Circular regions of same sizes were selected for each dot, and by using multi-regional background subtraction the corrected signal was quantified as background-corrected sum. Relative signal intensities were calculated by dividing each background corrected signal coming from one lipid drop by the sum of corrected signals from PI, PI(4)P, PI(4,5)P_2_ and PI(3,4,5)P_3_ or from PI and all phosphorylated derivatives (Membrane Lipid Strip™ or PIP Strip™, Echelon Biosciences).

### Liposome-binding assays

All lipids used in the assay were purchased from Avanti® Polar Lipids, Inc. The liposome-binding assays were performed as described in Julkowska et al. (2013) with the modifications that sonication (10% amplitude for 1 min, 3 s on, 20 s off, Digital Sonifier® W-250 D, Branson Ultrasonic Corporation) was used for liposome formation instead of extrusion, and 250 mM sucrose was contained in the extrusion buffer (25 mM Tris–HCl pH 7.5, 1 mM DTT) instead of raffinose. 1,2-dioleoyl-*sn*-glycero-3-phosphocholine (PC), 1,2-dioleoyl-*sn*-glycero-3-phosphoethanolamine (PE), 1,2-dioleoyl-*sn*-glycero-3-phospho-(1′-myo-inositol) (PI), 1,2-dioleoyl-*sn*-glycero-3-phospho-(1′-myo-inositol-4′-phosphate) (PI(4)P) and 1,2-dioleoyl-*sn*-glycero-3-phospho-(1′-myo-inositol-4′,5′-bisphosphate) (PI(4,5)P_2_) were each dissolved 20:9:1 in chloroform:methanol:water, mixed to a total of 300 nmol with 7% PI, and pelleted by vacuum centrifugation. The resulting pellet was rehydrated in extrusion buffer and sonicated, and then extruded over a 0.2 µm polycarbonate membrane using a mini-extruder (Avanti®). Liposomes were harvested at 50,000×*g* for 15 min and suspended in 25 µl binding buffer. The size and homogeneity of liposome samples was checked by dynamic light scattering using a Zetasizer Nano S (Malvern Panalytical). 12 pmol of protein in 25 µl of binding buffer were incubated with the 25 µl liposomes for 30 min at room temperature. Supernatant (s) and pelleted membrane fractions (m) were separated by centrifugation at 18,000×*g* (30 min) and both analyzed by SDS-PAGE followed by immunoblot using the α-PATL2-1 IgG antibody and detection, as described above. Band intensities were quantified using the Multiplex Band Analysis feature of AlphaView®Software (ProteinSimple). Relative membrane fraction intensity was calculated in % as signal in the liposome fraction (m) versus the 100% total signal (s + m). For statistical analysis, data were processed using analysis of variance (ANOVA) and Fisher’s least significant difference post hoc test.

## Results

### Complexity of the SEC14L-PITP family expanded during land plant evolution

We retraced the SEC14L-PITPs in the green lineage by evaluating the number of proteins and their domain composition. Sequences were recovered from selected species of green algae as well as plants, representing different evolutionary stages of the green lineage. Unicellular *Chlamydomonas reinhardtii* and multicellular *Volvox carteri*, both belong to *Chlorophyta*, have a low degree of cell differentiation. In the representatives of this ancestral green algae taxon, we identified six and eleven single-domain SEC14 proteins (SEC14-only proteins) (Figs. S1A, S1B). In most of them, a CTN domain is associated with the SEC14 domain. This resembles the organization of the yeast SEC14-PITP family. *Charophyta* have a complex morphology, are evolutionarily advanced and among closest living algal relatives of land plants. The charophytic algal model *Chara braunii* has six SEC14-only proteins, one of them devoid of the CTN, and one multi-domain SEC14L-PITP (Fig. S1C). The latter, (CBR_g38007), has a N-terminal plant homeodomain (PHD) and a DNA-binding homeobox-DDT domain along with WHIM motifs 1 and 2 involved in packing the DDT domain (Aravind and Iyer 2012). Such a combination was not identified in any other species investigated here.

Liverworts count as most ancient Bryophytes and first land plants. Eleven SEC14L-PITPs were found in the liverwort *Marchantia polymorpha* (Fig. S1D). Seven were SEC14-only proteins, three with and four without CTN. The remaining three proteins were SEC14-GOLD domain proteins, similar to Arabidopsis PATLs. The lycophyte *Selaginella moellendorffii*, an ancient vascular plant, has 14 SEC14L-PITPs (Fig. S2A). Ten of them are Sec14-only proteins, one without CTN. Three are SEC14-GOLD proteins. We found one new multidomain protein with an N-terminal domain similar to the human Ganglioside-induced differentiation-associated protein 2 (GDAP2) domain. ADP-ribose is a potential substrate for the GDAP2 domain (Hassa et al. 2006; Martzen et al. 1999). The function of the protein is unknown, but could be required for developmental transitions occurring only in vascular plants.

**Fig. 1:**
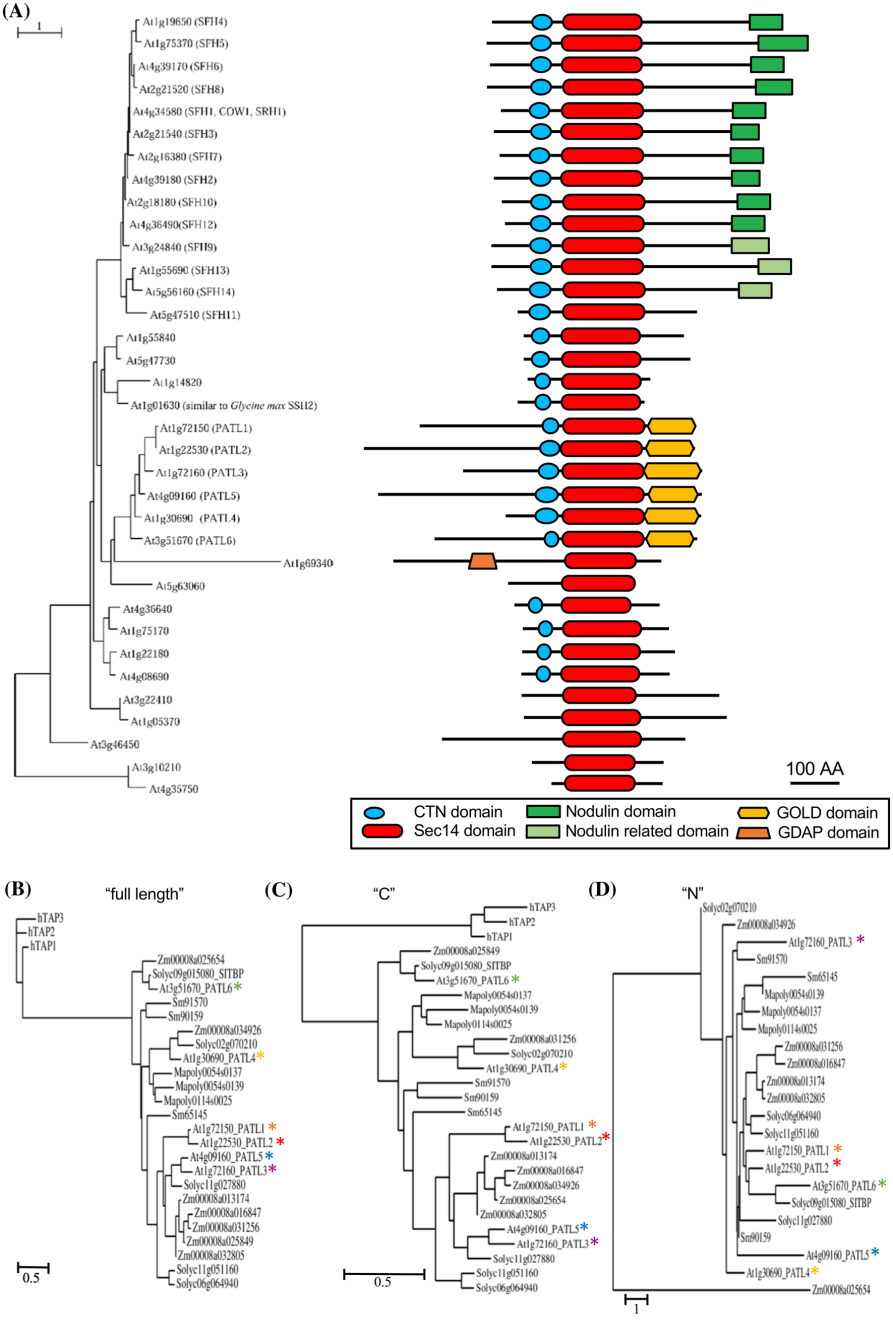
The flowering plant SEC14L-PITP superfamily is complex, and PATLs form three clades. **a** Phylogenetic relationships of *Arabidopsis thaliana* SEC14L-PITPs. Left, phylogenetic tree, generated with full-length amino acid sequences. Right, modular structure of SEC14L-PITPs; different domains, represented in different colors, as indicated. **b**–**d** Phylogenetic analysis of green lineage SEC14-GOLD proteins, generated with **b** full-length (“full-length”), **c** the CTN-SEC14-GOLD regions (“C”), **d** the variable N-termini (“N”). Three human TAP proteins represented outgroups in **b**, **c**. Size bars of phylogenetic trees represent the numbers of substitutions per amino acid position, as indicated

In Arabidopsis, we identified two new (At3g10210 and At4g35750) SEC14L-PITPs, summing up to 35 proteins (Mousley et al. 2007) (Fig. 1a). 15 of them are SEC14-only proteins, nine with a CTN. 20 proteins are multi-domain proteins, including the six PATLs with an additional GOLD domain, one with an additional GDAP domain and 13 SEC14L-PITPs with a nodulin or nodulin-related domain. In other distantly related flowering plants, such as the eudicot tomato (*Solanum lycopersicum*, *Solanaceae*) and the monocot maize (*Zea mays*, *Poaceae*), 32 and 54 SEC14L-PITPs are present, comprising 11 and 20 multi-domain proteins, again with either GOLD, GDAP, nodulin or nodulin-related domains (Figs. S2B, S3). The plant-specific SEC14-nodulin protein family is only present in investigated flowering plants (Denance et al. 2014; Kapranov et al. 2001; Vincent et al. 2005). Some of these SEC14-nodulin proteins were shown to function as general regulators in polarizing membrane trafficking (Ghosh et al. 2015; Huang et al. 2013; Vincent et al. 2005).

Taken together, the numbers and modular complexities of SEC14L-PITPs increased with the developmental complexity of multicellular eukaryotes in the green lineage. The increasing complexity is likely linked with new functions acquired to coordinate plant development with environmental constraints on dry land.

### Plant SEC14-GOLD (PATL) proteins divide into three clades based on sequence comparisons

Plant multi-domain SEC14L-PITPs group in similar branches of the phylogenetic trees, in contrast to SEC14-only proteins, that are scattered in different branches (Fig. 1a; Figs. S1, S2, S3). Three distinct PATL clades were suggested from Arabidopsis-tomato PATL comparisons, namely AtPATL6/SITBP, AtPATL4 and AtPATL1/2/3/5 (Bermudez et al. 2018), and hypothesized to mediate different cellular functions (Peterman et al. 2006). Phylogenetic analysis with all land plant PATL protein sequences, identified in this work, either using full length or CTN-SEC14-GOLD (termed here “C”) sequences (Fig. 1b, c), confirms the existence of three clades. The AtPATL1/2/3/5 clade is only present in vascular plants, while the AtPATL6 clade is exclusive to flowering plants. The AtPATL4 clade, closer to AtPATL1/2/3/5 than AtPATL6 clade, is most related to the ancient moss PATLs. The N-terminal regions (termed here “N”), without CTN-SEC14-GOLD domains, have a low degree of sequence similarity (Fig. S4). N regions alone are not sufficient to construct a meaningful phylogenetic tree (Fig. 1d). N stretches are also absent in the human SEC14-GOLD α-tocopherol-associated proteins (TAPs) (Fig. 1d; Fig. S5).

Sequence diversification into three clades suggests that PATLs might fulfill diverse functions within the plant, perhaps associated with canonical roles (via CTN-SEC14-GOLD) and adaptive responses (via N).

### Expression analysis of Arabidopsis *PATL* genes confirms functional diversification into three groups

**Fig. 2:**
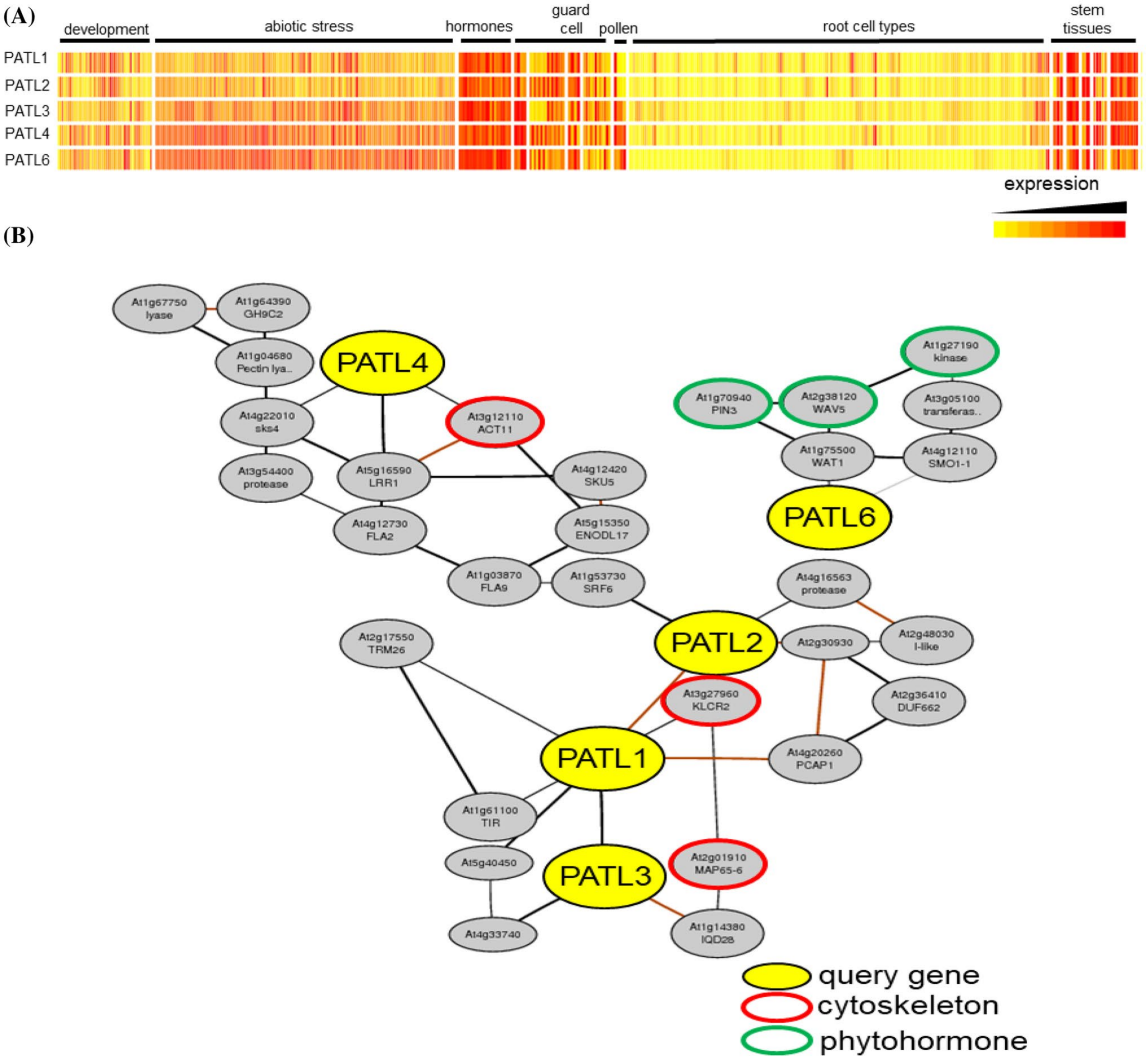
Arabidopsis *PATL* genes form three subgroups, based on gene expression profiles. **a** Heatmap showing expression patterns of *PATL* genes across development, tissues, environmental and hormone responses. **b** Genome-wide co-expression networks of *PATL* genes with auxin response and cytoskeleton genes. No data are available for *PATL5*

Available transcriptome data for five of the six Arabidopsis *PATL* genes reflect the three different clades (Fig. 2a). Generally, all genes are more highly expressed in stem than root tissues, and regulated under the influence of plant hormones and during development. Particularly *PATL4* and *PATL6* are responsive to abiotic stresses. *PATL1* and *PATL2* are closely co-regulated, which supports the suggestion that they may have arisen from gene duplication events (Peterman et al. 2004). *PATL4* is associated in a distant way with the *PATL1*, *PATL2*, *PATL3* coexpression cluster (Fig. 2b). Within this coexpression cluster, four genes encode proteins related to actin, kinesin motor proteins and microtubules, suggesting that *PATL1*, *PATL2*, *PATL3* and *PATL4* function might be related to cytoskeleton organization and dynamics (Fig. 2b; Table S1). *PATL6* is part of a different coexpression cluster with an enrichment of auxin-related genes (Fig. 2b; Table S1).

Partially overlapping expression patterns and co-expression suggest that PATL family genes participate in common processes and may therefore exhibit partial functional redundancy. Despite of that, some specific functions are noted, consistent with the observed sequence diversification of PATL clades.

### The CTN-SEC14 and GOLD domains of PATL2 protein confer different specificities in phosphoinositide binding

**Fig. 3:**
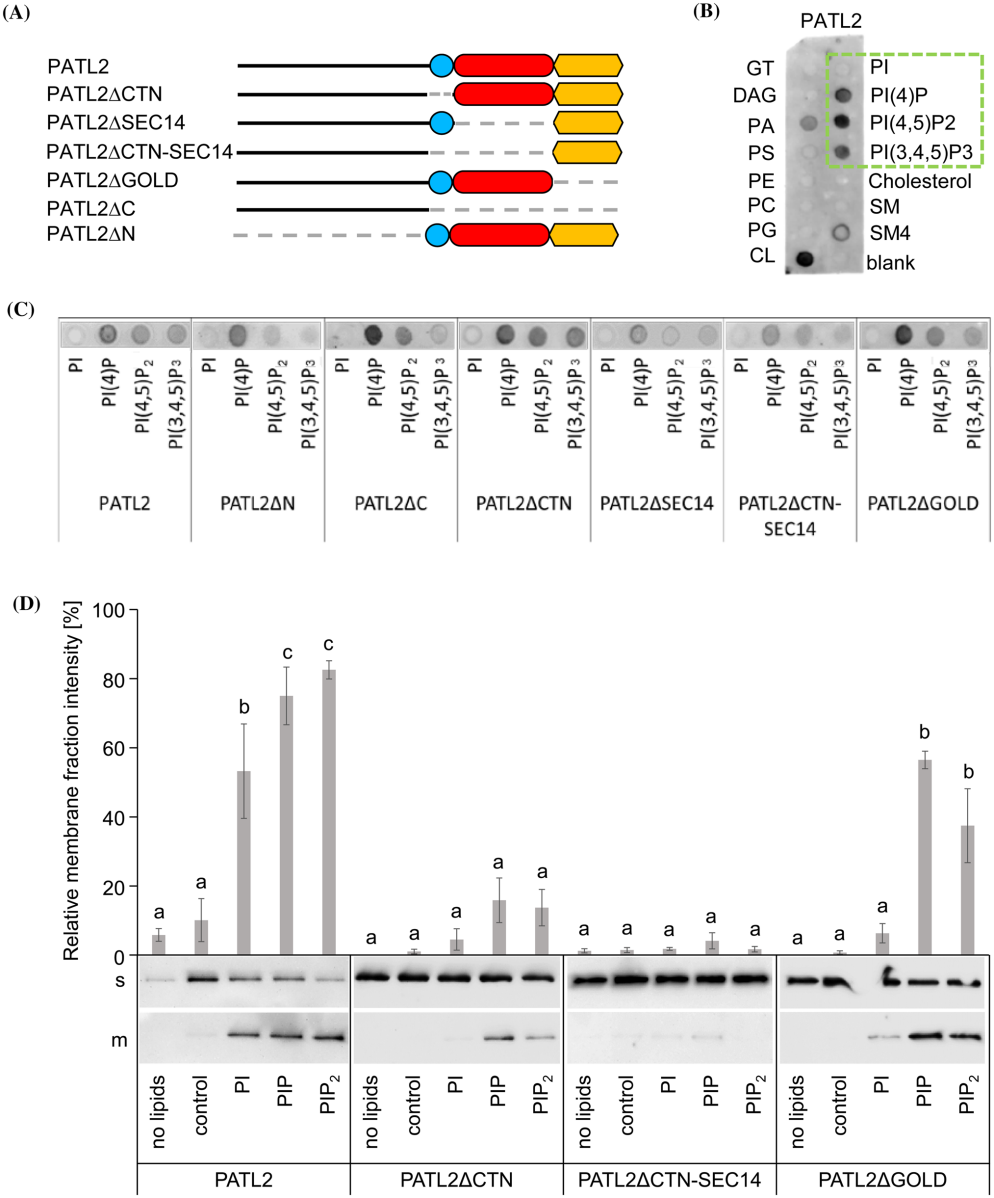
Phosphoinositide binding of PATL2 is dependent on CTN-SEC14 and GOLD domains. **a** Schematic representation of PATL2 deletion mutants. **b**, **c** StrepII-PATL2 protein–lipid overlay assay. Green rectangle in **b**, phosphoinositide binding signal which is presented for the different PATL2 derivatives in **c**. **d** Immunoblot of liposome-binding assays of StrepII-PATL2 and selected deletion mutants, associated with soluble (s) and membrane (m) fractions. No lipids, indicates controls without liposomes; control, indicates PC/PE-based backbone liposome controls; additional supplementation of liposomes with PI (PI); PI(4)P (PIP); PI(4,5)P_2_, (PIP_2_); note the gel migration behavior of proteins (see Figure S6C). The bar diagram shows relative abundance of proteins associated with membrane (m) fractions in % of the total protein abundance of soluble (s) and m fractions. Bars represent mean ± SD (n = 3). Different letters indicate statistically significant differences (ANOVA and Tukey HSD post hoc test, p < 0.05)

The CTN-SEC14 domain is the canonical element of SEC14L-PITPs for lipid transfer functions and this domain associates with the membrane. The GOLD domain is essential for PATL3 recruitment to the PM via EXO70A1 (Wu et al. 2017). However, detailed knowledge is lacking about the roles of the different domains of SEC14-GOLD proteins in plants. We used PATL2 from Arabidopsis to dissect the roles of PATL2 domains in membrane attachment in vitro. Deletion mutants lacking domains were generated (Fig. 3a). The PM and cell plate localization of full-length PATL2 and the ability to bind PIPs in protein-lipid overlay assays (Suzuki et al. 2016; Tejos et al. 2017) raised the question whether specific PIPs are recognized by individual PATL2 domains. However, all PATL2 deletion versions assayed were able to bind to any type of immobilized PIPs using a lipid strip, including those devoid of CTN, SEC14 and GOLD domains (Fig. 3b, c). Cardiolipin, sulfatide and phosphatidic acid were also bound, presumably because of their negative charges, mimicking PIPs (Fig. 3b). Using a specific PIP strip, PATL2 and all deletion proteins bound any type of PIPs. Mostly, however, PI(3)P was preferred, followed by either PI(5)P or PI(3,5)P_2_ (Figs S6A, S6B). Since PI(3,4,5)P_3_ is likely not produced in plant cells (Munnik and Nielsen 2011) this lipid species was not part of our further investigation. Overlay assays show binding to immobilized phospholipid units, while liposome assays provide information of binding to phospholipids in the context of a synthetic membrane. The latter mimics better PIP recognition in a biological membrane environment.

To conduct a liposome assay, PI(4)P and PI(4,5)P_2_ were chosen since they are predominantly present at the PM and the cell plate (Simon et al. 2014) where PATL2 was found to be localized (Suzuki et al. 2016; Tejos et al. 2017). Basic liposomes prepared with PC/PE served as controls, and neither PATL2 protein nor any mutant version associated with them in the membrane fractions (Fig. 3d; membrane fractions designated m). If PI was added to these liposomes, a low amount of PATL2 protein was found in the liposome membrane fraction, but no significant increase was noted for the three tested PATL2 mutants, PATL2∆CTN, PATL2∆CTN-SEC14 and PATL2∆GOLD (Fig. 3d). Addition of PI(4)P or PI(4,5)P_2_ increased the abundance of PATL2 and PATL2∆GOLD in the membrane fractions, although not to the same level for PATL2∆GOLD than for wild type, but again not of PATL2∆CTN or PATL2∆CTN-SEC14 (Fig. 3d). This shows that the N and GOLD domains are not sufficient for liposome and PIP binding, and that CTN or CTN-SEC14 domains are required. All protein versions were detected in all supernatant fractions, showing that they were recognized by antisera (Fig. 3d; supernatant fractions designated s). Hence, the CTN-SEC14 domain is required for association with PIPs in the membrane, while the GOLD domain steers additionally PIP membrane targeting of PATL2.

### The GOLD domain of SEC14-GOLD protein PATL2 specifies membrane binding in plant cells

**Fig. 4:**
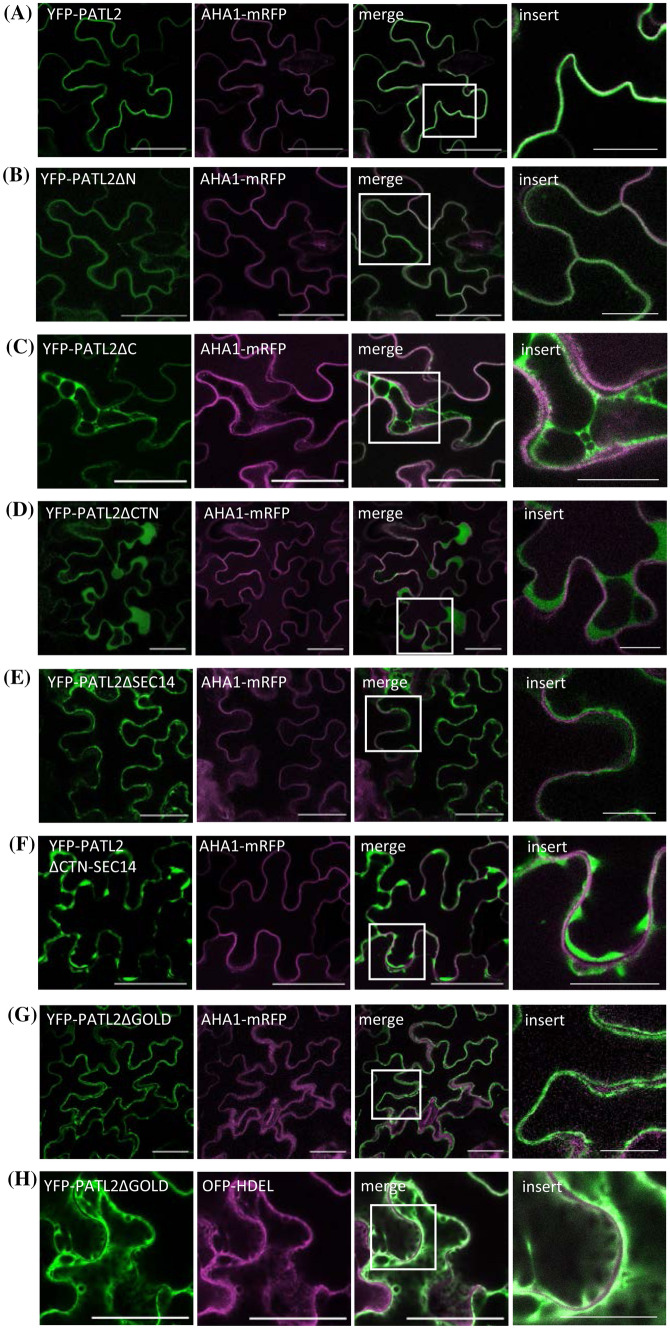
Cellular localization of PATL2 is specified by the CTN-SEC14 and GOLD domains. **a**–**h** Co-localization of YFP-PATL2 or its deletion mutants (green) with either PM marker AHA1-mRFP or ER marker HDEL-mOFP (magenta), in tobacco epidermis cells. Size bars 50 µm. The insert image shows an enlargement of the zone marked in the merge image. Size bars for the insert images 20 µm

The PATL2-liposome association raised the question of the role of PATL2 domains in a cellular context. When expressed in plant cells, full-length YFP-PATL2 was present mainly at the PM, co-localizing with the PM marker AHA1 (Fig. 4a). Deletion of PATL2-N had no effect on the PM localization (Fig. 4b), indicating that the N region has different significance for the protein. Deletion of the CTN-SEC14 module (PATL2∆C, PATL2∆CTN, PATL2∆SEC14 and PATL2∆CTN-SEC14 combinations) abolished membrane association, and the proteins were primarily present in the cytosol (Fig. 4c–f). This is consistent with the observed inability of PATL2∆CTN-SEC14 to bind to liposomes (this work). A critical role of the CTN domain in the membrane association of SEC14L-PITPs (Fig. 4d) had previously been observed (Skinner et al. 1993). Deletion of the GOLD domain abolished PM association, and instead, led to localization at the ER membrane, marked by OFP-tagged HDEL (Fig. 4g, h). The observation is consistent with the role of the GOLD domain in binding PI(4,5)P_2_-containing liposomes (this work) and the PM, enriched in PI(4,5)P_2_ (Mamode Cassim et al. 2019).

Taken together, the CTN-SEC14 domain is essential for PATL2 membrane association, however, the GOLD domain carries information for PM targeting. The N region, not involved in membrane targeting, may confer physiological tasks.

## Discussion

In this work, we present a phylogenetic analysis of the SEC14L-PITP superfamily in the green lineage and show that the complexity of SEC14L-PITPs increased during land plant evolution. We dissect the modular domain structure of a SEC14-GOLD protein, Arabidopsis PATL2, and demonstrate that the individual domains of PATL2 contribute differently to lipid binding and membrane association.

The numbers of family members and the modular configurations of SEC14L-PITPs increased during land plant evolution, similar as in the animal lineage. This reflects certainly the high versatility of SEC14L-PITP roles in multicellular organisms, where developmental and environmental specifications of functions become increasingly connected, as highlighted by the case of SEC14-GOLD proteins in Arabidopsis. *Charaphyceae* have some land plant features, related to the function of SEC14-GOLD proteins. Their cell divisions involve formation of a phragmoplast with presence of specific proteins, and a subset of auxin functions seem present, as inferred from the partial presence of auxin signaling and PIN-mediated auxin transport genes in Chara (Nishiyama et al. 2018). Thus, it is surprising that SEC14-GOLD proteins are lacking in Chara, since these proteins are involved in cell plate formation and auxin responses in higher plants like Arabidopsis (Peterman et al. 2004; Tejos et al. 2017). Perhaps SEC14-GOLD proteins were acquired in land plants to integrate cell division and auxin responses with complex environmental response networks, more important on dry land. The fact that PATL1/PATL2/PATL3 and PATL4 are present in one common co-expression network suggests that they might take a role in regulating cytoskeleton function. This corresponds well to the known involvement of PATL1 and PATL2 in cell division (Peterman et al. 2004; Suzuki et al. 2016), a process dependent on the cytoskeleton. Enrichment of auxin-related genes in PATL6 co-expression analysis is consistent with the function of PATLs in auxin-dependent PIN1 localization (Tejos et al. 2017). Additionally, analysis of multiple *patl* mutants in Arabidopsis demonstrated a redundant function and critical role of the protein family in polarity and patterning (Suzuki et al. 2016). Taken together, SEC14-GOLD proteins are partly redundant in land plants, while some specific functions of each are noted.

Besides the SEC14 domain, many SEC14 proteins also have the CTN, some even a GOLD domain, and only plant SEC14-GOLD proteins additionally an N region. PATL2 is composed of a discrete N region and a C region with the conserved CTN-SEC14 and GOLD domains. We investigated the contributions of individual domains to membrane binding using deletion mutants of full domains. Because the respective remaining domains were entities on their own, we are confident that domain deletions did not affect the protein structure in a manner that the functions of individual remaining domains were perturbed.

The C regions with CTN-SEC14-GOLD domains of different SEC14-GOLD proteins do not differ much in their amino acid sequence (Peiro et al. 2014; Peterman et al. 2004). PATL2 binds to the PM, and the C region is sufficient for PM-binding. Consistently, the localization of PATL2ΔN did not differ from that of PATL2, while deletion of the C region (PATL2ΔC) resulted in cytosolic localization of the protein. Deletion of only the CTN, SEC14 or both domains (PATL2ΔCTN, PATL2ΔSEC14, PATL2ΔCTN-SEC14) also resulted in cytosolic localization of the protein, consistent with the inability of PATL2∆CTN and PATL2∆CTN-SEC14 to bind PI(4)P and PI(4,5)P_2_-loaded liposomes. The canonical CTN-SEC14 domain might attach to phosphate groups of PIPs through surface-located conserved basic amino acids, two of these are present in PATL2 (R373; K542) (Fig. S5) (Kono et al. 2013). The importance of the CTN-SEC14 domain is also consistent with literature data. The CTN domain of yeast Sec14p adds membrane-binding capabilities to a predominantly cytosolic rat SEC14L-PITP (Skinner et al. 1993). Moreover, a point mutation in the SEC14 domain of human p50RhoGAP leads to loss of protein-membrane association (Sirokmany et al. 2006), similar as the deletion of the CTN-SEC14 domain of human PTP-MG2 (Saito et al. 2007b). Hence, the conserved function of the CTN-SEC14 domain in membrane-binding also holds true for plant proteins.

The GOLD domain is present in the single-domain protein p24 and in multi-domain proteins. In the latter, the GOLD domain co-occurs with additional domains, having a function in binding membrane lipids and proteins (Anantharaman and Aravind 2002; Sohda et al. 2001). The GOLD domain of PATL2 adds specificity for PM recognition and for binding to liposomes containing PI(4)P and PI(4,5)P_2_, presumably due to a reported conserved lysine repeat. It may specifically bind PI(4,5)P_2_, since it aligns well to the PI(4,5)P_2_-binding motif of two proteins, AP180 and µ2-adaptin, which are involved in clathrin-coated vesicle formation (Fig. S4) (Ford et al. 2001; Mao et al. 2001; Peterman et al. 2004; Rohde et al. 2002). Deletion of the GOLD domain (PATL2ΔGOLD) shifts binding from the PM to the endomembrane system, and lowers binding to liposomes containing PI(4,5)P_2_ or PI(4)P. The above-mentioned amino acid similarities among C regions of SEC14-GOLD proteins suggest conserved domain functions for them (Anantharaman and Aravind 2002; Ford et al. 2001; Mao et al. 2001; Peiro et al. 2014; Peterman et al. 2006; Saito et al. 2007a; Simon et al. 2014; Skinner et al. 1993).

The N regions of SEC14-GOLD proteins are unique to the plant kingdom and are not present in human SEC14-GOLD counterparts. Furthermore, they vary in every plant SEC14-GOLD protein. The sequence diversity of the N region suggests rapid evolution and unique function. The N region of PATL2 does not mediate liposome binding, if the CTN-SEC14 domain is lacking (PATL2ΔCTN-SEC14). Also in the cellular context, the N region alone lacking the CTN-SEC14-GOLD module (PATL2ΔC), does not bind membranes. However, the N region confers binding to immobilized phospholipid units, and we explain this with charges and ionic interactions involving the positively-charged [(K)KE(E); (EE)EK] repeats of PATL2-N. Perhaps the N regions of SEC14-GOLD proteins contribute to membrane interactions in cellular contexts by binding to membrane proteins, such as shown in the case of PATL1 binding the salt transporter SOS1 via the N region (Zhou et al. 2018). Thus, the N regions might confer specific functions to SEC14-GOLD proteins and be relevant for specific physiological responses.

In the future, it will be important to understand the molecular mechanisms underlying the PATL functions, modulated by their domains and by specific protein–protein and protein–lipid interactions.

## Electronic supplementary material

Below is the link to the electronic supplementary material.

Supplementary file1 (PDF 1331 kb)

## Abbreviations

CaM4: Calmodulin-4
CTN: CRAL-TRIO-N-terminal extension
GOLD: Golgi dynamics
PATL: PATELLIN
PE: Phosphatidylenthanolamine
PC: Phosphatidylcholine
PI: Phosphatidylinositide
PIP: Phosphatidylinositol phosphate
PITP: Phosphatidylinositol transfer protein
PM: Plasma membrane
