# Supplementary material for: Phylogenetic analysis of plant multi-domain SEC14-like phosphatidylinositol transfer proteins and structure–function properties of PATELLIN2
Source: Plant Mol Biol. 2020 Sep 11;104(6):665–78. doi: 10.1007/s11103-020-01067-y (PMC7674337; doi:10.1007/s11103-020-01067-y)
Supplement: Supplementary file 1 — Supplementary file1 (PDF 1331 kb) [file 11103_2020_1067_MOESM1_ESM.pdf]

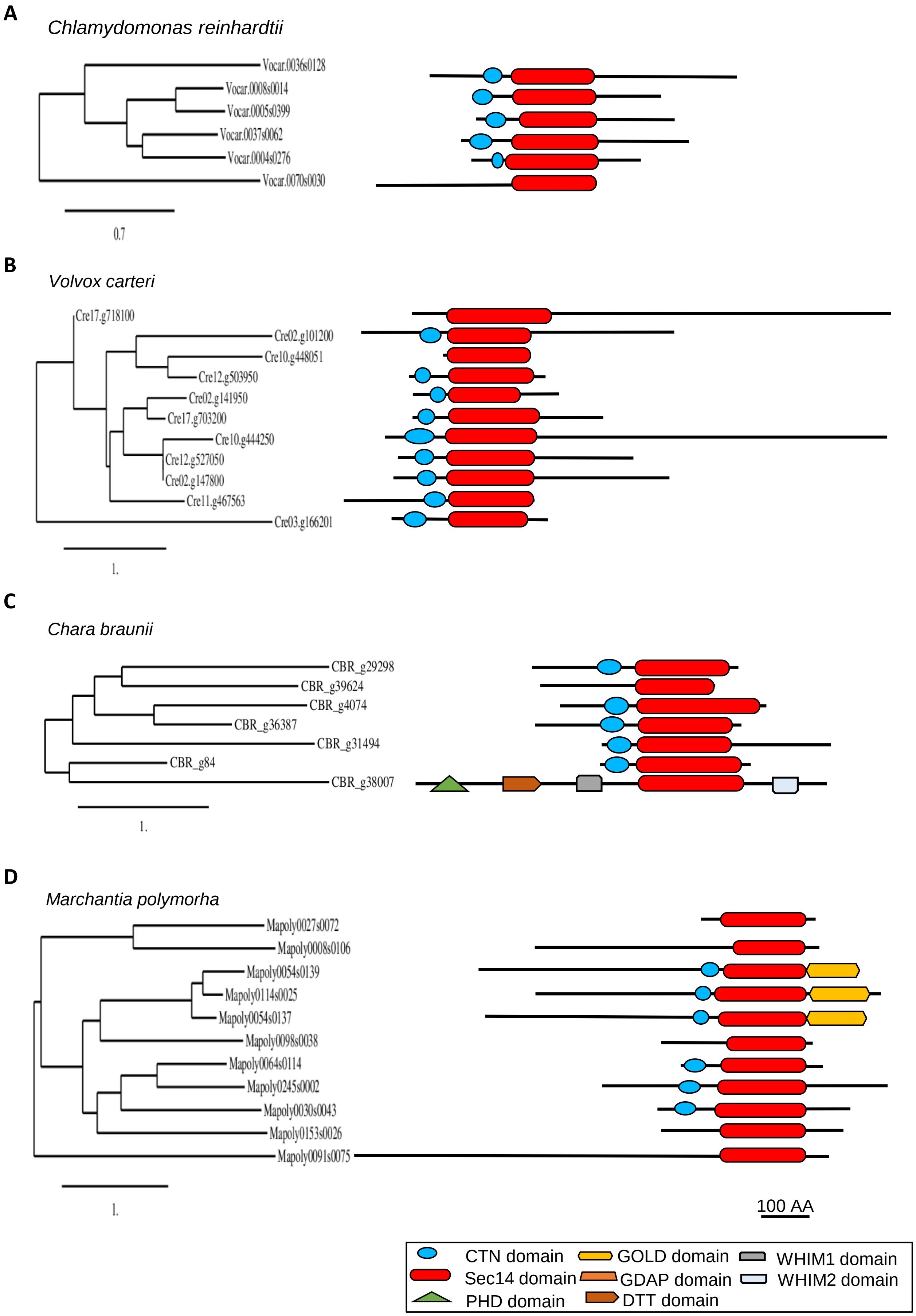

A
Chlamydomonas reinhardtii
Vocar.0036s0128
Vocar.0008s0014
Vocar.0005s0399
Vocar.0037s0062
Vocar.0004s0276
Vocar.0070s0030
0.7
B
Volvox carteri
Cre17.g718100
Cre02.g101200
Cre10.g448051
Cre12.g503950
Cre02.g141950
Cre17.g703200
Cre10.g444250
Cre12.g527050
Cre02.g147800
Cre11.g467563
Cre03.g166201
1.
C
Chara braunii
CBR_g29298
CBR_g39624
CBR_g4074
CBR_g36387
CBR_g31494
CBR_g84
CBR_g38007
1.
D
Marchantia polymorha
Mapoly0027s0072
Mapoly0008s0106
Mapoly0054s0139
Mapoly0114s0025
Mapoly0054s0137
Mapoly0098s0038
Mapoly0064s0114
Mapoly0245s0002
Mapoly0030s0043
Mapoly0153s0026
Mapoly0091s0075
1.
100 AA
CTN domain
Sec14 domain
PHD domain
GOLD domain
GDAP domain
DTT domain
WHIM1 domain
WHIM2 domain

Supplemental Figure 1. Phylogenetic analysis and modular structure of the SEC14L-PITP superfamilies in green algae and liver moss.

Left, Phylogenetic trees, generated with full-length amino acid sequences. Right, modular structures of SEC14L-PITPs. (A) *Chlamydomonas reinhardtii*; (B) *Volvox carteri*; (C) *Chara braunii*; (D) *Marchantia polymorha.* Different domains are represented in different color, as indicated. Protein primary structure and relative length of domains are drawn to scale.

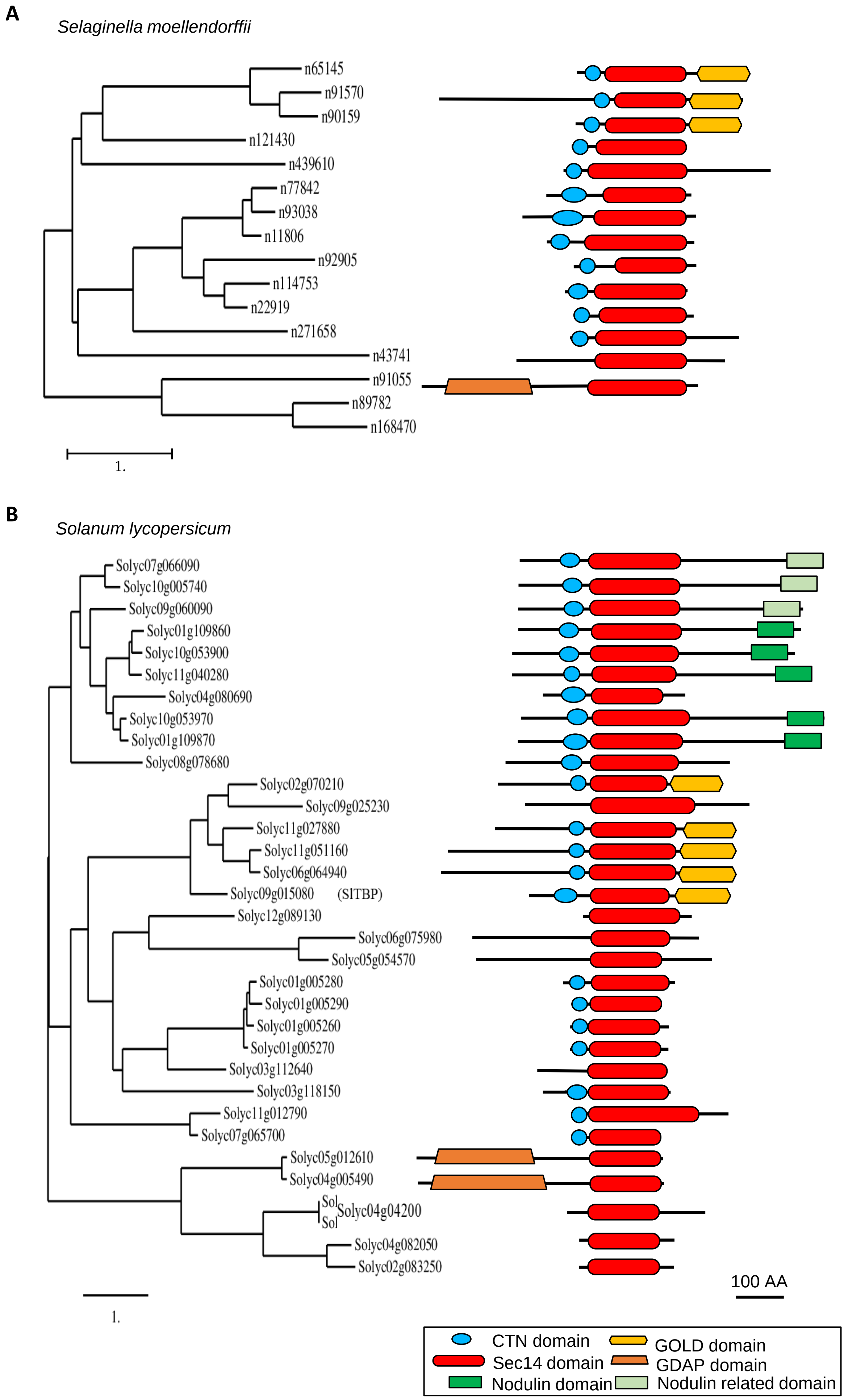
A
Selaginella moellendorffii
n65145
n91570
n90159
n121430
n439610
n77842
n93038
n11806
n92905
n114753
n22919
n271658
n43741
n91055
n89782
n168470
1.
B
Solanum lycopersicum
Solyc07g066090
Solyc10g005740
Solyc09g060090
Solyc01g109860
Solyc10g053900
Solyc11g040280
Solyc04g080690
Solyc10g053970
Solyc01g109870
Solyc08g078680
Solyc02g070210
Solyc09g025230
Solyc11g027880
Solyc11g051160
Solyc06g064940
Solyc09g015080 (SlTBP)
Solyc12g089130
Solyc06g075980
Solyc05g054570
Solyc01g005280
Solyc01g005290
Solyc01g005260
Solyc01g005270
Solyc03g112640
Solyc03g118150
Solyc11g012790
Solyc07g065700
Solyc05g012610
Solyc04g005490
Solyc04g04200
Solyc04g082050
Solyc02g083250
1.
100 AA
CTN domain
Sec14 domain
Nodulin domain
GOLD domain
GDAP domain
Nodulin related domain

Supplemental Figure 2. Phylogenetic analysis and modular structure of the SEC14L-PITP superfamilies in a lycophyte and eudicot tomato.

Left, Phylogenetic tree, generated with full-length amino acid sequences. Right, modular structures of SEC14L-PITPs. (A) *Selaginella moellendorffii*; (B) *Solanum lycopersicum.* Different domains are represented in different color, as indicated. Protein primary structure and relative length of domains are drawn to scale.

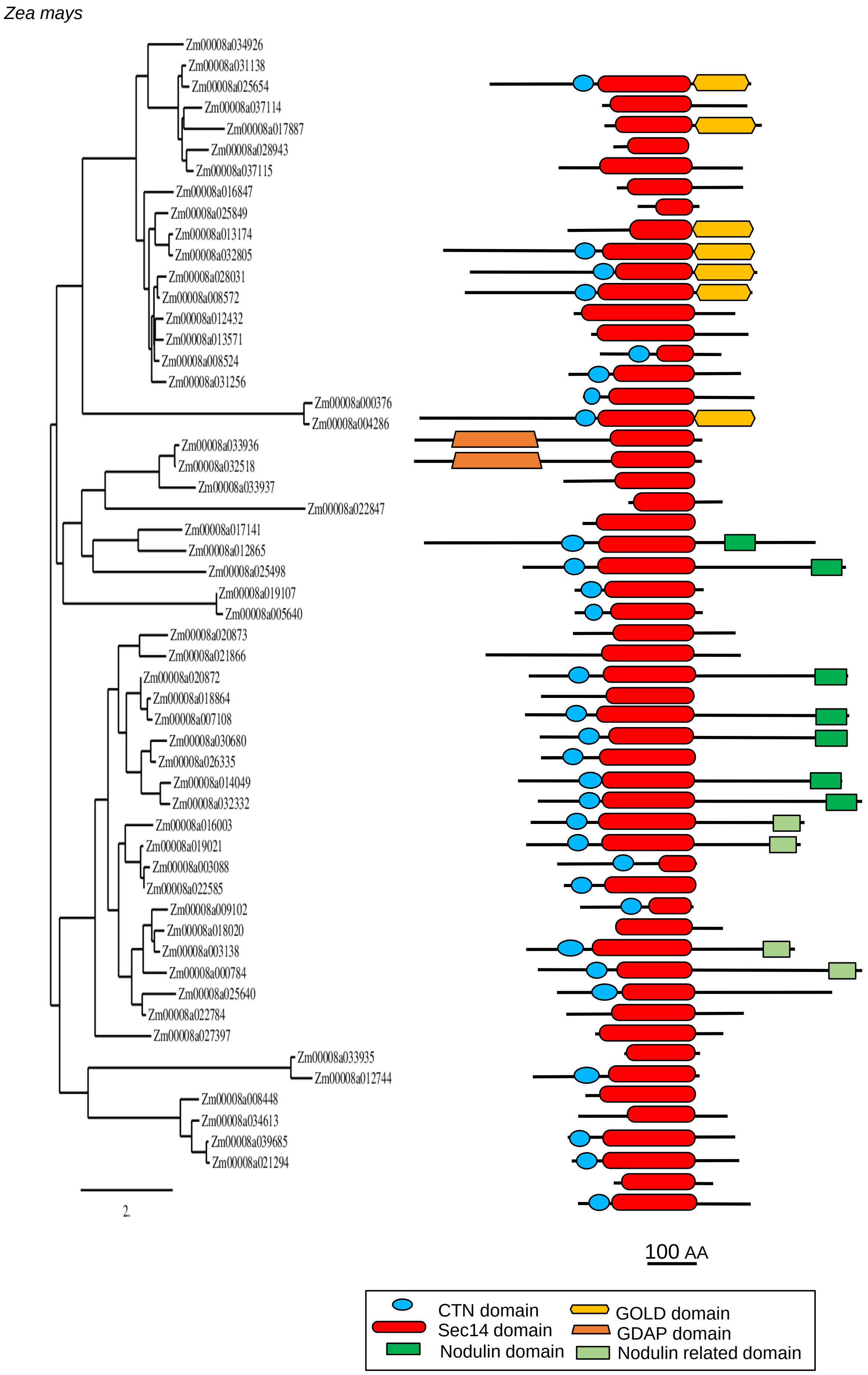

Zea mays
Zm00008a034926
Zm00008a031138
Zm00008a025654
Zm00008a037114
Zm00008a017887
Zm00008a028943
Zm00008a037115
Zm00008a016847
Zm00008a025849
Zm00008a013174
Zm00008a032805
Zm00008a028031
Zm00008a008572
Zm00008a012432
Zm00008a013571
Zm00008a008524
Zm00008a031256
Zm00008a000376
Zm00008a004286
Zm00008a033936
Zm00008a032518
Zm00008a033937
Zm00008a022847
Zm00008a017141
Zm00008a012865
Zm00008a025498
Zm00008a019107
Zm00008a005640
Zm00008a020873
Zm00008a021866
Zm00008a020872
Zm00008a018864
Zm00008a007108
Zm00008a030680
Zm00008a026335
Zm00008a014049
Zm00008a032332
Zm00008a016003
Zm00008a019021
Zm00008a003088
Zm00008a022585
Zm00008a009102
Zm00008a018020
Zm00008a003138
Zm00008a000784
Zm00008a025640
Zm00008a022784
Zm00008a027397
Zm00008a033935
Zm00008a012744
Zm00008a008448
Zm00008a034613
Zm00008a039685
Zm00008a021294
2.
100 AA
CTN domain
Sec14 domain
Nodulin domain
GOLD domain
GDAP domain
Nodulin related domain

Supplemental Figure 3. Phylogenetic analysis and modular structure of the SEC14L-PITP superfamily in monocot maize (*Zea mays*).

Left, Phylogenetic tree, generated with full-length amino acid sequences. Right, modular structures of SEC14L-PITPs. Different domains are represented in different color, as indicated. Protein primary structure and relative length of domains are drawn to scale.

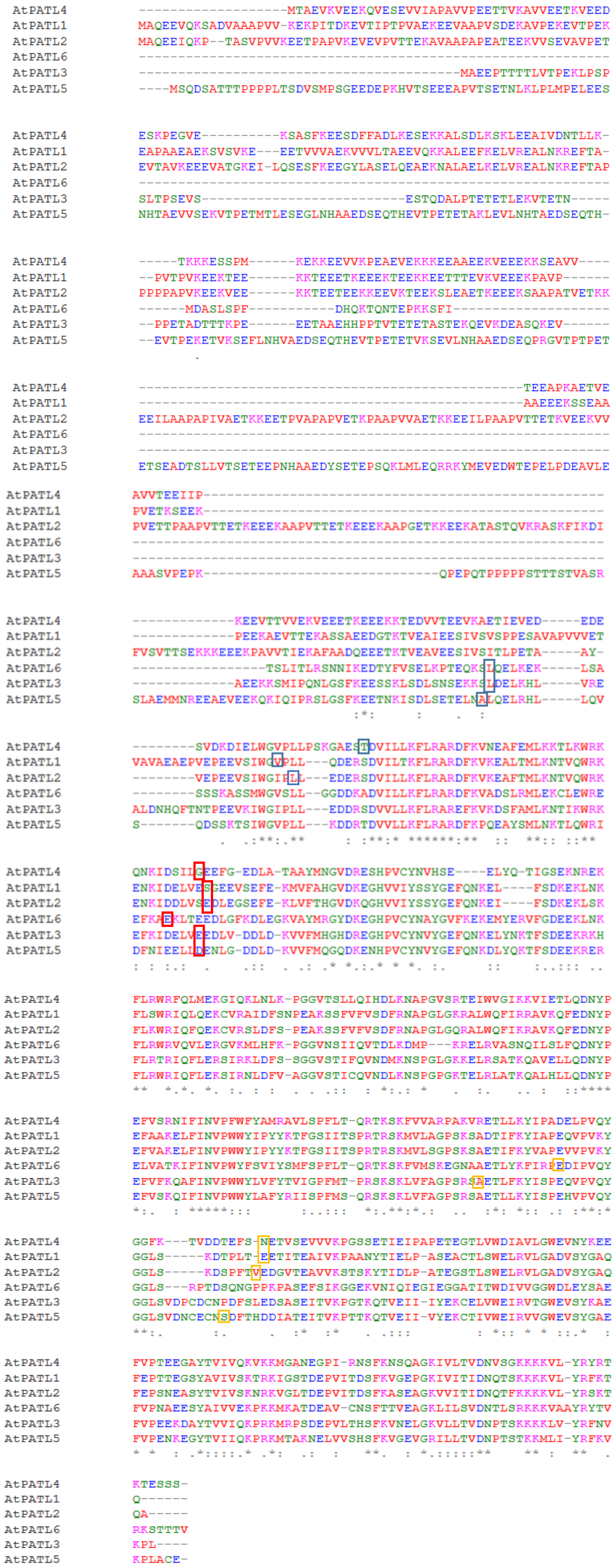

Supplemental Figure 4. Alignment of Arabidopsis PATL protein sequences. The start of different domains is indicated by a box, in blue, CTN, in red, SEC14, in yellow, GOLD domain. The colors of amino acid letters represent red, hydrophobic; green, polar uncharged; pink, basic; blue, acidic amino acids.

```
AT1G22530      MAQEEIQKPTASVPVVKEETPAPVKEVEVPVTTEKAVAAPAPEATEEKVVSEVAVPETEV  60
TAP3           ------------------------------------------------------------  0
TAP1           ------------------------------------------------------------  0
TAP2           ------------------------------------------------------------  0

AT1G22530      TAVKEEEVATGKEILQSESFKEEGYLASELQEAEKNALAELKELVREALNKREFTAPPPP  120
TAP3           ------------------------------------------------------------  0
TAP1           ------------------------------------------------------------  0
TAP2           ------------------------------------------------------------  0

AT1G22530      PAPVKEEKVEEKKTEETEEKKEEVKTEEKSLEAETKEEEKSAAPATVETKKEEILAAPAP  180
TAP3           ------------------------------------------------------------  0
TAP1           ------------------------------------------------------------  0
TAP2           ------------------------------------------------------------  0

AT1G22530      IVAETKKEETPVAPAPVETKPAAPVVAETKKEEILPAAPVTTETKVEEKVVPVETTPAAP  240
TAP3           ------------------------------------------------------------  0
TAP1           ------------------------------------------------------------  0
TAP2           ------------------------------------------------------------  0

AT1G22530      VTTETKEEEKAAPVTTETKEEEKAAPGETKKEEKATASTQVKRASKFIKDIFVSVTTSEK  300
TAP3           ------------------------------------------------------------  0
TAP1           ------------------------------------------------------------  0
TAP2           ------------------------------------------------------------  0

                                                                       ⬇
AT1G22530      KKEEEKPAVVTIEKAFAADQEEETKTVEAVEESIVSITLPETAAYVEPEEVSIWGIPLLE  360
TAP3           -----------------------------MSSR-VGDLSPQQQEALARFRENLQDLLPIL  30
TAP1           -----------------------------MSGR-VGDLSPRQKEALAKFRENVQDVLPAL  30
TAP2           ------------------------------------------------------------  0

AT1G22530      DERSDVILLKFLRARDFKVKEAFTMLKNTVQWRKENKIDDLVSEDLEGSE-FEKLVFTHG  419
TAP3           PNADDYFLLRWLRARNFDLQKSEDMLRRHMEFRKQQDLDNIVTWQPPEVIQLYDSGGLCG  90
TAP1           PNPDDYFLLRWLRARSFDLQKSEAMLRKHVEFRKQKDIDNIISWQPPEVIQQYLSGGMCG  90
TAP2           -----------------------------MEFRKTMDIDHILDWQPPEVIQKYMPGGLCG  31
                                            :::**  .:*.::  :              *

AT1G22530      VDKQGHVVIYSSYGEFQNKEIFSDKEKLSKFLKWRIQFQEKCVRSLDFSPE-AKSSFVFV  478
TAP3           YDYEGCPVYFNIIGSLDPKGLLLSASKQD-MIRKRIKVCELLLHECELQTQKLGRKIEMA  149
TAP1           YDLDGCPVWYDIIGPLDAKGLLFSASKQD-LLRTKMRECELLLQECAHQTTKLGRKVETI  149
TAP2           YDRDGCPVWYDIIGPLDPKGLLFSVTKQD-LLKTKMRDCERILHECDLQTERLGKKIETI  90
                * :*  * :.  * :: * :: .  * . ::: :::  *  ::.   .      ..

AT1G22530      SDFRNAPGLGQRALWQFIKRAV----KQFEDNYPEFVAKELFINVPWWYIPYYKTFGSII  534
TAP3           LMVFDMEGLSLKHLWKPAVEVYQQFFSILEANYPETLKNLIVIRAPKLFPVAFNLVKSFM  209
TAP1           TIIYDCEGLGLKHLWKPAVEAYGEFLCMFEENYPETLKRLFVVKAPKLFPVAYNLIKPFL  209
TAP2           VMIFDCEGLGLKHFWKPLVEVYQEFFGLLEENYPETLKFMLIVKATKLFPVGYNLMKPFL  150
                 . :  **. : :*:   ..       :* **** :   :.:..   :   :: .  ::

AT1G22530      TSPRTRSKMVLSGPSKSAETIFKYVAPEVVPVKYGGLSKDSPFTV---------------  579
TAP3           SEE-TRRKIVILGD-NWKQELTKFISPDQLPVEFGGTMTDPDGNPKCLTKINYGGEVPKS  267
TAP1           SED-TRKKIMVLGA-NWKEVLLKHISPDQVPVEYGGTMTDPDGNPKCKSKINYGGDIPRK  267
TAP2           SED-TRRKIIVLGN-NWKEGLLKLISPEELPAQFGGTLTDPDGNPKCLTKINYGGEIPKS  208
               :.  ** *:::  *  :  : : * ::*: :*.::**  .*   .

AT1G22530      ---EDGVTEA------VVKSTSKYTIDLPATEGSTLSWELRVLGADVSYGAQFEPSNEAS  630
TAP3           YYLCEQVRLQYEHTRSVGRGSSLQVENEILFPGCVLRWQFASDGGDIGFGVFLKTKMGEQ  327
TAP1           YYVRDQVKQQYEHSVQISRGSSHQVEYEILFPGCVLRWQFMSDGADVGFGIFLKTKMGER  327
TAP2           MYVRDQVKTQYEHSVQINRGSSHQVEYEILFPGCVLRWQFSSDGADIGFGVFLKTKMGER  268
                   : *         : :.:*  .       *..* *::   *.*:.:*  :: .

AT1G22530      -----YTVIVSKNR-KVGLTDEPVITDSFKASEAGKVVITIDNQTF--KKKKVLYRSKTQ  682
TAP3           QSAREMTEVLPSQRYNAHMV---PEDGSLTCLQAGVYVLRFDNTYSRMHAKKLSYTVEVL  384
TAP1           QRAGEMTEVLPNQRYNSHLV---PEDGTLTCSDPGIYVLRFDNTYSFIHAKKVNFTVEVL  384
TAP2           QRAGEMTDVLPSQRYNAHMV---PEDGNLTCSEAGVYVLRFDNTYSFVHAKKVSFTVEVL  325
                     * :: .:* :  :.      ..:.. : *  *: :**     : **: :  :.

AT1G22530      A---------------------      683
TAP3           LPDKASEETLQSLKAMRPSPTQ      406
TAP1           LPDKASEEKMKQLGAGTPK---      403
TAP2           LPDEGMQKYDKELTPV------      341
```

Supplemental Figure 5. Alignment of AtPATL2 with human TAP protein sequences.
The start of the CTN-SEC14-GOLD region is indicated by an arrow. The colors of amino acid letters representred, hydrophobic; green, polar uncharged; pink, basic; blue, acidic amino acids.

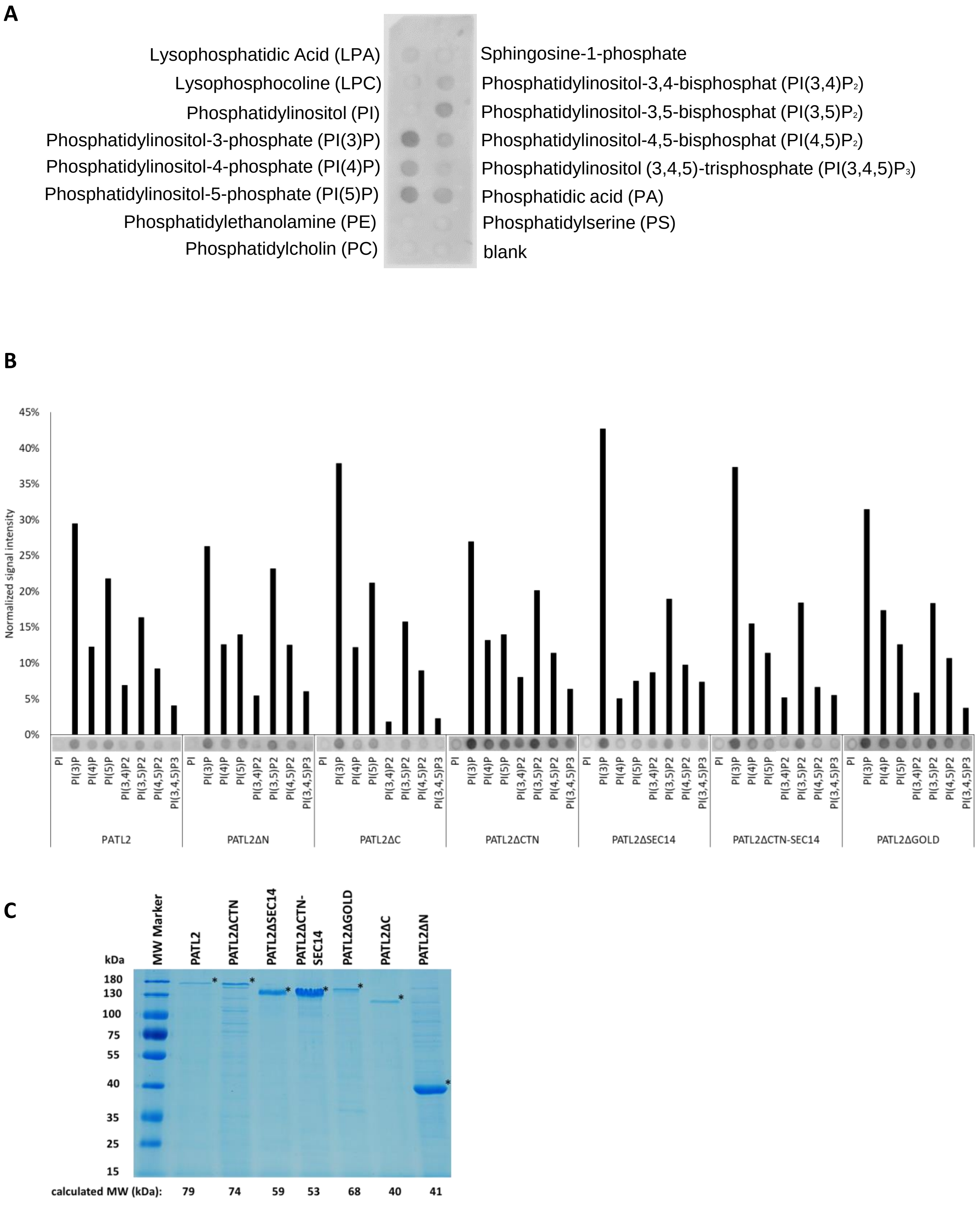

Supplemental Figure 6. Protein–phosphoinositide overlay assay and electrophoretic migration behavior of StrepII-PATL2 and deletion mutants.

(A, B) StrepII-PATL2 and deletion mutant protein–phospholipid overlay assay. PIP binding is quantified in (B). (C) Electrophoretic migration behavior of StrepII-PATL2 and deletion mutant proteins, analyzed by in SDS-PAGE, followed by Coumassie staining. StrepII-PATL2 and deletion mutant protein bands are indicated by an asterisk. The theoretical masses of StrepII-PATL2 and deletion mutants are indicated below. In the presence of the N-terminal domain, migration of proteins is drastically retarded, presumably due to the acidic character of the N-terminal part, as reported before (Peterman et al., 2004; Armstrong and Roman, 1993).

Supplemental Table S1. List of genes in coexpression modules

AGI number	Name	description
**The genes in *PATL1-4* coexpression module**		
At1g72150	PATL1	PATELLIN1
At1g22530	PATL2	PATELLIN2
At1g72160	PATL3	PATELLIN3
At2g17550	TRM26	TON1 RECRUITING MOTIF 26
At1g61100		disease resistance protein
At5g40450	RBB1	REGULATOR OF BULB BIOGENESIS1
At4g33740		MYB-like transcriptoion factor
At3g27960	KLCR2	KINESIN LIGHT CHAIN-RELATED 2
At2g01910	MAP65-6	Microtubule-associated protein
At1g14380	IQD28	IQ-DOMAIN 28
At4g16563		aspartyl protease
At2g30930		hypothetical protein
At4g20260	PCAP1	PLASMA-MEMBRANE ASSOCIATED CATION-BINDING PROTEIN 1
At2g48030		DNAse-I-like
At2g36410	DUF662	transcriptional activator
At1g64390	GH9C2	GLYCOSYL HYDROLASE 9C2
At1g53730	SRF6	STRUBBELIG RECEPTOR FAMILY6
At1g30690	PATL4	PATELLIN4
At3g12110	ACT11	ACTIN11
At1g03870	FLA9	FASCICLIN-LIKE ARABINOOGALACTAN 9
At5g16590	LRR1	LEUCINE RICH REPEAT PROTEIN 1
At1g67750		Pectate lyase
At4g12730	FLA2	FASCICLIN-LIKE ARABINOGALACTAN 2
At4g22010	SKS4	SKU5 SIMILAR4
At1g04680		Pectin lyase-like
At3g54400		Aspartyl protease
At5g15350	ENODL17	EARLY NODULIN-LIKE PROTEIN 17
At4g12420	SKU5	protein of unknown function involved in directed root tip growth
**The genes in *PATL6* coexpression module**		
At1g27190	BIR3	BAK1-INTERACTING RECEPTOR-LIKE KINASE 3
At1g70940	PIN3	PIN-FORMED 3
At2g38120	WAV5 (AUX1)	AUXIN RESISTANT 1
At3g05100	transferase	SAM methyl transferase
At1g75500	WAT1	WALLS ARE THIN 1 transporter
At4g12110	SMO1-1	STEROL-4ALPHA-METHYL OXIDASE 1-1
At3g51670	PATL6	PATELLIN6

Supplemental Table S2. Information on the generation of PATL2 deletion mutants.

PATL2 deletion mutant	AA position	Predicted molecular weight (kDa)	predicted molecular weight stepII-tagged (kDa)	Molecular running weight stepII-tagged (kDa)
PATL2	1-683	76	79	~ 170
PATL2ΔN	358-683	38	41	~ 40
PATL2ΔCTN	1-357; 403-683	71	74	~ 170
PATL2ΔSEC14	1-402; 577-684	56	59	~ 160
PATL2ΔCTN-SEC14	1-357; 577-685	50	53	~ 160
PATL2ΔGOLD	1-579	65	68	~ 120
PATL2ΔC	1-357	37	40	~ 160

Supplemental Table S3. Primers used in this study.

Primer	Sequence (5'-3')
PATL2B1F _N	GGGGACAAGTTTGTACAAAAAAGCAGGCTTTATGGCTCAAGAAGAGATACAG
PATL2B2stopR	GGGGACCACTTTGTACAAGAAAGCTGGGTTTATGCTTGGGTTTTGGACC
PATL2ΔGOLDstopB2	GGGGACCACTTTGTACAAGAAAGCTGGGTTTAAACGGTGAATGGAC
PATL2ΔSEC14_F	ATCGACGACCTAGTCTTCACCGTTGAAGAT
PATL2ΔSEC14_R	ATCTTCAACGGTGAAGACTAGGTCGTCGAT
PATL2ΔCTN-SEC14_F	ATCTGGGGAATCCCATTCACCGTTGAAGAT
PATL2ΔCTN-SEC14_R	ATCTTCAACGGTGAATGGGATTCCCCAGAT
PATL2ΔCTN_F	CAATCTGGGGAATCCCATCAGAAGATCTTGA
PATL2ΔCTN_R	TCAAGATCTTCTGATGGGATTCCCCAGATTG
PATL2ΔCstopB2	GGGGACCACTTTGTACAAGAAAGCTGGGTTTATGGGATTCCCCAGATTGAGAC
PATL2ΔNB1_N	GGGGACAAGTTTGTACAAAAAAGCAGGCTTTCTTCTAGAGGACGAAAGATCC
PATL2_F_BamHI	AGGAGTGGATCC T ATGGCTCAAGAAGAGATACAG
PATL2_R_NotI	AGGAGTGCGGCCGC TTA TGCTTGGGTTTTGGACC
PATL2ΔGOLD_R_NotI	AGGAGTGCGGCCGC TTA AACGGTGAATGGACTATC
PATL2ΔN_F_BamHI	AGGAGTGGATCC T CTTCTAGAGGACGAAAGATCC
PATL2ΔC_R_NotI	AGGAGTGCGGCCGC TTA TGGGATTCCCCAGATTG
